# Mortality following Hip Fracture in Men with Prostate Cancer

**DOI:** 10.1371/journal.pone.0074492

**Published:** 2013-09-27

**Authors:** Mieke Van Hemelrijck, Hans Garmo, Karl Michaëlsson, Andreas Thorstenson, Olof Akre, Pär Stattin, Lars Holmberg, Jan Adolfsson

**Affiliations:** 1 King's College London, School of Medicine, Division of Cancer Studies, Cancer Epidemiology Group, London, United Kingdom; 2 Institute for Environmental Medicine, Karolinska Institute, Stockholm, Sweden; 3 Regional Cancer Centre, Uppsala Örebro, Uppsala, Sweden; 4 Department of Surgical Sciences, Section of Orthopaedics, Uppsala University, Uppsala, Sweden; 5 Department of Molecular Medicine and Surgery, Karolinska Institute, Stockholm, Sweden; 6 Department of Surgery, Section of Urology, Capio S: t Görans Hospital, Stockholm, Sweden; 7 Clinical Epidemiology Unit, Karolinska Institute, Stockholm, Sweden; 8 Department of Surgical and Perioperative Sciences, Urology and Andrology, Umeå University, Umeå, Sweden; 9 Department of Surgery, Urology Service, Memorial Sloan-Kettering Cancer Center, New York, New York, United States of America; 10 Department of Surgical Sciences, Uppsala University, Uppsala, Sweden; 11 CLINTEC Department, Karolinska Insititutet, Stockholm, Sweden; Roswell Park Cancer Institute, United States of America

## Abstract

**Background:**

Hip fractures are associated with increased mortality and are a known adverse effect of androgen deprivation therapy (ADT) for prostate cancer (PCa). It was our aim to evaluate how mortality after hip fracture is modified by PCa and ADT.

**Methods:**

PCa dataBase Sweden (PCBaSe 2.0) is based on the National PCa Register and also contains age and county-matched PCa-free men. We selected all men (n = 14,205) who had been hospitalized with a hip fracture between 2006 and 2010; 2,300 men had a prior PCa diagnosis of whom 1,518 (66%) were on ADT prior to date of fracture. Risk of death was estimated with cumulative incidence and standardized mortality ratios (SMRs) to make comparisons with the entire PCa population and the general population.

**Results:**

Cumulative incidences indicated that there was a higher risk of death following a hip fracture for PCa men on ADT than for PCa men not on ADT or PCa-free men, particularly in the first year. The SMRs showed that PCa men on ADT with a hip fracture were 2.44 times more likely to die than the comparison cohort of all PCa men (95%CI: 2.29-2.60). This risk was especially increased during the first month (5.64 (95%CI: 4.16–7.48)). In absolute terms, hip fractures were associated with 20 additional deaths per 1,000 person-years in PCa men not on ADT, but 30 additional deaths per 1,000 person-years for PCa men on ADT, compared to all PCa men.

**Conclusion:**

Hip fractures are associated with higher all-cause mortality in PCa men on ADT than in PCa men not on ADT or PCa-free men, especially within the first three months.

## Introduction

Androgen deprivation therapy (ADT) is standard treatment for metastatic prostate cancer (PCa) and is also increasingly used after PSA relapse in men who have undergone curative treatment and who have a long life expectancy [1]. There is a risk of adverse effects after ADT, including rapid loss of bone-mineral density occurring within the first six to twelve months. Androgens are involved in the stimulation of bone growth through the stimulation of growth hormone secretion [1], [2], [3]. In the population-based Prostate Cancer data Base (PCBaSe) Sweden, men with PCa treated with ADT had a higher risk of fractures than PCa men not on ADT [4].

In the general population hip fractures are associated with risk of mortality [5], [6], [7], [8]. A prospective cohort study of men and women aged 60 years and older found that all low-trauma fractures were associated with increased mortality risk for five to ten years [9]. A meta-analysis based on 22 cohort and 17 case-control studies showed that older adults have a five to eight-fold increased risk for all-cause mortality during the first three months after a hip fracture [10].

Few population-based observational studies have investigated mortality following hip fractures among men with PCa [11], [12], [13], [14], [15], [16]. In the most recent study, using SEER-Medicare data, men with bone metastasis and skeletal-related events were 10 times more likely to die than men with no bone metastasis during a median follow-up of three years [16].

Following the existing evidence for increased mortality after a hip fracture in the general population and the increased risk of hip fractures among PCa men on ADT, we investigated whether having PCa and treatment with ADT modifies the known association between hip fracture and mortality. This could help identify whether prevention of these hip fractures would result in improving survival for PCa men on ADT. We used nationwide population-based cohort data [17] to assess the effect of PCa and ADT on the link between hip fractures and risk of death while taking into account disease risk category and comorbidities.

## Methods

### Study population and data collection

**Figure 1:**
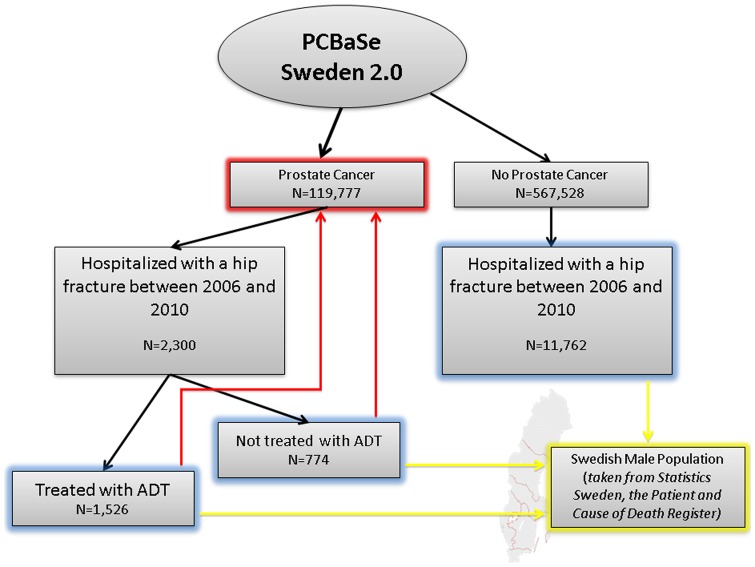
Selection of study cohort from PCBaSe 2.0. The effect of hip fracture on mortality is studied in three groups (blue boxes): men free of PCa, PCa men not on ADT, and PCa men on ADT. The effects are quantified using standardized mortality rates (SMRs) so that all three groups (blue boxes) are compared to the Swedish male population (yellow arrows). To take into account the effect of PCa on mortality, the two PCa groups are also compared to all men diagnosed with PCa, as registered in PCBaSe (red arrows).

In 2010, the National Prostate Cancer Register (NPCR) of Sweden was linked to a number of other population-based registers via the use of the Swedish personal identity number [18]. The resulting database, PCBaSe Sweden 2.0, also includes a control-series of men free of PCa at the time of sampling. The controls were matched by county of residence and birth year with an index case [18]. From the 119,777 men with PCa and their control series in PCBaSe 2.0, we selected all men (n = 14,205) who had been hospitalized with a first hip fracture (ICD-10: S720, S721, S722), as registered in the National Patient Register [19] between 2006 and 2010. A total of 2,300 men were previously diagnosed with PCa of whom 1,526 were treated with ADT (blue boxes in Figure 1). Androgen deprivation therapy prior to the hip fracture was defined by bilateral orchiectomy, as registered in the National Patient Register, or filed prescriptions for GnRH agonists in the Prescribed Drug Register. Men who had received primary curative treatment and received prescriptions for GnRH agonists for <120 days, according to the Defined Daily Dose, i.e. a neoadjuvant use, were not included in the study group. Following our previous paper on risk of fractures following ADT [20], we did not include any other types of ADT as GnRH agonists and orchiectomy were the most common type of ADT in our study population.

The main exposure in this study was hip fracture classified as non-pathological, whereas the main outcome was death as registered in the National Cause of Death Register [19]. We included a list of potential confounders such as PCa risk category, history of fractures, civil status, and Charlson Comorbidity Index (CCI). From the NPCR, which started in 1996 and captures more than 96% of all newly diagnosed, biopsy-confirmed PCas compared to the Swedish Cancer Registry [17], we have detailed information about tumour characteristics [17], [21]. Prostate cancer risk categories were defined according to a modification of the National Comprehensive Cancer Network Guidelines [18], [22]. Information about previous fractures as well as CCI was taken from the National Patient Register [4]. Previous fractures were grouped as follows: wrist (ICD-10: S524–526), upper arm (ICD-10: S422), and vertebral column (ICD-10: S22 and 32). The CCI was calculated to assess the burden of concomitant disease at time of hip fracture and consists of 18 groups of diseases with a specific weight assigned to each disease category (1, 2, 3, and 6). Given the special interest in PCa in this study, PCa was not included in the CCI, but reported separately. The weights are then summed to obtain an overall score, resulting in the three comorbidity levels of the index: 0 for no comorbidity, 1 for mild and 2+ for severe comorbidity [23]. Information on GnRH agonists was taken from the Prescribed Drug Register, which includes all prescriptions dispensed in Swedish pharmacies from July 2005 [24]. Also information on bisphosphonates, drugs given to prevent bone loss and to treat osteoporosis, was taken from this Register. Follow-up was defined from time of hip fracture until time of death, emigration, or end of study (31^st^ December 2011), whichever occurred first.

### Analysis

Since we aim to identify whether PCa and/or ADT (potential effect modifier) modify the known association between hip fractures (main exposure) and risk of death (main outcome), all our analyses focused on the following three cohorts: (1) PCa men on ADT, (2) PCa men not on ADT, and (3) PCa-free men (blue boxes in Figure 1). We graphically represented the risk of death following a hip fracture using cumulative incidences for these three cohorts of interest.

Consistent with our previous analysis on risk of fractures in men with PCa [4], we then calculated standardized mortality ratios (SMRs). Firstly, we calculated SMRs by comparing the observed risk of death following a hip fracture in (1) PCa men on ADT, (2) PCa men not on ADT, and (3) PCa-free men, with the risk of death in the total Swedish male population (yellow arrows in Figure 1). These SMR calculations took into account age (1-year classes going from 50 to 100+) and year of follow-up (1-year classes going from 2006 to 2011) and were done to confirm the already known association between hip fracture and risk of death. Based on calculations using the formulas by Jones and Swerdlow [25], the general population rates cause very little bias in the SMRs [26].

Since men with PCa have an additional risk of death given their cancer, we then calculated SMRs to compare the observed risk of death following a hip fracture in PCa men with or without ADT with the risk of death in all men with PCa in PCBaSe (red arrows in Figure 1). These SMR calculations were matched on time since PCa diagnosis (0–3; 3–6; 6–9; 9–12, and 12+ years), age (5-year classes going from 50–54, 55–59, ., 85–90, 90+), PCa risk category at time of diagnosis (low risk, intermediate risk, high risk, regionally metastatic/locally advance, distant metastasis [18]) and time since start of ADT (orchiectomy: no, 0–5; 5–10; 10+ years or GnRH agonists: no; 0–2; 2–4; 4+ years). The latter SMRs were calculated in an attempt to identify whether the effects of PCa and/or ADT on the association between hip fracture and mortality were observed due to ADT, the cancer, or patient frailty. These SMRs thus took into account disease severity.

Hence, the SMRs were calculated by comparing observed number of deaths in those with hip fractures with the expected number in the background population [27], [28]. The 95% CIs for SMRs were estimated assuming that the observed cases had a Poisson distribution using Byar's normal approximation [29], [30]. Bisphosphate use was not used in any of the above calculations as its prescription was not common practice yet during the study period resulting in less than 4% of our study population being on this drug.

Finally, to address how PCa and/or ADT may modify the association between hip fracture and risk of death we also calculated the absolute risk differences in the three groups described above while using the Swedish male population as the reference cohort (yellow arrows in Figure 1). Again, we also calculated the absolute risk of death following a hip fracture for PCa men with and without ADT while using all men with PCa in PCBaSe as the reference cohort (red arrows in Figure 1).

Statistical analyses were performed with Statistical Analysis Systems (SAS) release 9.2 (SAS Institute, Cary, NC). The Swedish Central Ethics Committee (Dnr Ö 14–2007) and the Ethics Committee at Umeå University (Dnr 07–049M) have approved the project.

## Results

Of all men with hip fractures, a total of 2,300 were previously diagnosed with PCa of whom 1,518 (66%) were on ADT. Detailed descriptive statistics of the study population are shown in Table 1. Bisphosphonates was taken by about 3% of men with PCa not on ADT and 4% of men with PCa on ADT (Table 1).

**Table 1:** Baseline characteristics of men hip fractures between 2006 and 2010 in PCBaSe 2.0.

	PCa, ADT (n = 1526)		PCa, no ADT (n = 774)		No PCa (n = 11762)		All study subjects (n = 14062)	
**Age group, n (%)**								
50–75	157	(10.3)	152	(19.6)	1764	(15.0)	2073	(14.7)
75–79	255	(16.7)	143	(18.5)	1821	(15.5)	2219	(15.8)
80–84	456	(29.9)	200	(25.8)	3043	(25.9)	3699	(26.3)
85–89	444	(29.1)	171	(22.1)	3349	(28.5)	3964	(28.2)
90+	214	(14.0)	108	(14.0)	1785	(15.2)	2107	(15.0)
**Risk category, n (%)**								
Low risk	84	(5.5)	233	(30.1)			317	(2.3)
Intermediate risk	257	(16.8)	219	(28.3)			476	(3.4)
High risk	606	(39.7)	198	(25.6)			804	(5.7)
Regionally metastatic	194	(12.7)	28	(3.6)			222	(1.6)
Distant metastases	358	(23.5)	46	(5.9)			404	(2.9)
Missing data	27	(1.8)	50	(6.5)			77	(0.5)
No PCa					11762	(100.0)	11762	(83.6)
**Previous fractures, n (%)**								
No fractures	1300	(85.2)	680	(87.9)	10261	(87.2)	12241	(87.1)
Wrist	51	(3.3)	15	(1.9)	259	(2.2)	325	(2.3)
Upper arm	42	(2.8)	16	(2.1)	322	(2.7)	380	(2.7)
Vertebral column	112	(7.3)	50	(6.5)	799	(6.8)	961	(6.8)
Combination	21	(1.4)	13	(1.7)	121	(1.0)	155	(1.1)
**Charlson Comorbidity Index, n (%)**								
0	507	(33.2)	254	(32.8)	3696	(31.4)	4457	(31.7)
1	385	(25.2)	188	(24.3)	2928	(24.9)	3501	(24.9)
2	290	(19.0)	133	(17.2)	2101	(17.9)	2524	(17.9)
3+	344	(22.5)	199	(25.7)	3037	(25.8)	3580	(25.5)
**Civil status, n(%)**								
Married	823	(53.9)	407	(52.7)	5601	(47.6)	6831	(48.6)
Not married	111	(7.3)	72	(9.3)	1359	(11.6)	1542	(11.0)
Divorced	161	(10.6)	97	(12.5)	1407	(12.0)	1665	(11.8)
Widower	431	(28.2)	197	(25.5)	3395	(28.9)	4023	(28.6)
**Bisphosponates, n (%)**	63	(4.1)	25	(3.2)	292	(2.5)	380	(2.7)

**Figure 2:**
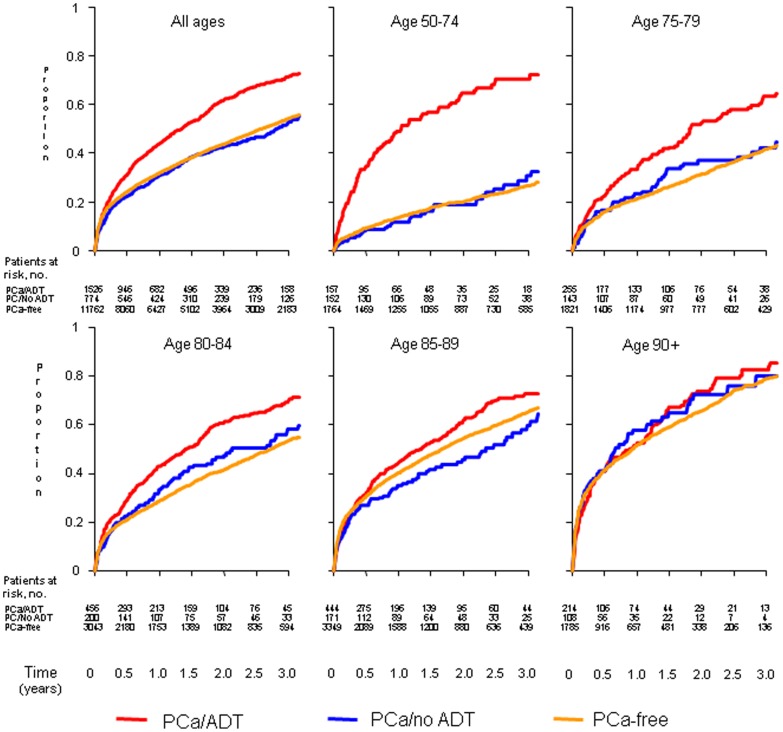
Cumulative incidence of death following a hip fracture among PCa-free men and PCa men with and without ADT, stratified by age at time of fracture.

The cumulative incidence figures (Figure 2) indicated that there was a higher risk of death following a hip fracture, particularly in the first year, for PCa men on ADT than for PCa men not on ADT or PCa-free men. The difference in mortality between the three cohorts was strongest in men aged 50–74 years and became weaker with increasing age, with almost no differences between the groups for men aged >90.

The SMRs comparing risk of death for PCa men with and without ADT as well as PCa-free men who experienced a hip fracture to the Swedish male population (yellow arrows in Figure 1) indicated a higher risk of overall death for PCa men on ADT (4.49 (95%CI: 4.20–4.78)) than for PCa men not on ADT (3.18 (95%CI: 2.85–3.53)) or PCa-free men (2.88 (95%CI: 2.81–2.96) (Results not shown). The related absolute risks for these comparisons showed that the absolute risk difference between PCa men on ADT with and without a hip fracture was 40 per 1,000 person-years, whereas a hip fracture would cause an additional 20 deaths per 1,000 person-years among PCa men not on ADT or PCa-free men (Results not shown).

These results indicated an increased risk of death following a hip fracture in each cohort studied, but since PCa itself is associated with an increased risk of death, it was necessary to redo the above analyses in which PCa men with and without ADT were compared to the entire PCa population (Table 2; Red arrows in Figure 1). Overall, the SMR was 2.44 (95%CI: 2.29–2.60) for PCa men on ADT and 2.97 (95%CI: 2.66–3.30) for PCa men not on ADT. However, within the first month after the hip fracture the SMR was 5.64 (95%CI: 4.16–7.48) and 5.53 (95%CI: 2.76–9.89) for men with and without ADT, respectively. Since these are relative comparisons and those on ADT also have more severe disease, the absolute risk differences are more clinically relevant (Table 2). A hip fracture would cause an additional 20 deaths per 1,000 person-years among PCa men not on ADT, but an additional 30 deaths per 1,000 person-years among PCa men on ADT (Table 2), compared to the total PCa population.

**Table 2:** SMR with 95% confidence intervals (95% CI) and absolute risk differences for mortality after hip fracture among PCa men on ADT and PCa men not on ADT.

	PCA ON ADT							PCA NOT ON ADT						
	RELATIVE RISK			ABSOLUTE RISK				RELATIVE RISK			ABSOLUTE RISKS			
	SMR	95% CI	Obs/Exp	Absolute risk for men on ADT with hip fracture	Absolute risk for men on ADT	Absolute Risk Difference	95% CI	SMR	95% CI	Obs/Exp	Absolute risk for men not on ADT with hip fracture	Absolute risk for men not on ADT	Absolute Risk Difference	95% CI
**Overall**	2.44	(2.29–2.60)	940/385.3	50.8	20.8	30.0	(26.7–33.2)	2.97	(2.66–3.30)	344/115.8	29.5	9.9	19.6	(16.5–22.7)
**Stratified by time since hip fracture**														
0-1 months	7.15	(6.14–8.27)	180/25.2	153.5	21.5	132.0	(109.6–154.4)	12.5	(9.78–15.6)	74/5.9	122.7	9.9	112.8	(84.9–140.8)
1-3 months	3.30	(2.86–3.78)	205/62.2	69.8	21.2	48.6	(39.0–58.1)	4.76	(3.73–6.00)	72/15.1	45.4	9.5	35.8	(25.4–46.3)
3-12 months	2.03	(1.79–2.31)	243/119.5	42.8	21.0	21.7	(16.3–27.1)	2.49	(1.97–3.10)	80/32.1	23.8	9.5	14.2	(9.0–19.4)
1-2 years	1.85	(1.60–2.13)	192/103.7	38.4	20.7	17.7	(12.2–23.1)	1.92	(1.47–2.46)	61/31.8	19.0	9.9	9.1	(4.3–13.8)
2+ years	1.61	(1.33–1.92)	120/74.7	32.4	20.1	12.2	(6.4–18.0)	1.85	(1.40–2.39)	57/30.8	19.8	10.7	9.1	(3.9–14.2)
**Stratified by CCI**														
0	2.13	(1.89–2.39)	289/135.4	43.1	20.2	22.9	(17.9–27.9)	1.72	(1.35–2.15)	76/44.3	16.5	9.6	6.9	(3.2–10.6)
1	2.29	(2.01–2.61)	229/99.9	48.0	20.9	27.1	(20.8–33.3)	2.72	(2.15–3.39)	78/28.7	26.8	9.9	16.9	(11.0–22.8)
2	2.76	(2.38–3.19)	190/68.7	55.4	20.0	35.4	(27.5–43.2)	3.51	(2.73–4.44)	69/19.7	37.2	10.6	26.6	(17.8–35.4)
3+	2.86	(2.50–3.25)	232/81.2	64.4	22.5	41.9	(33.6–50.2)	5.22	(4.33–6.23)	121/23.2	53.1	10.2	42.9	(33.5–52.4)
**Time since hip fracture for men with CCI = 0**														
0-1 months	5.64	(4.16–7.48)	48/8.5	121.0	21.5	99.6	(65.3–133.8)	5.53	(2.76–9.89)	11/2.0	53.5	9.7	43.9	(12.2–75.5)
1-3 months	2.72	(2.06–3.51)	58/21.4	56.5	20.8	35.7	(21.2–50.3)	3.88	(2.37–5.99)	20/5.2	35.7	9.2	26.5	(10.9–42.2)
3-12 months	1.93	(1.54–2.40)	81/41.9	40.1	20.7	19.4	(10.6–28.1)	1.78	(1.08–2.74)	20/11.3	16.2	9.1	7.1	(-0.0–14.2)
1-2 years	1.67	(1.29–2.14)	64/38.2	34.2	20.4	13.8	(5.4–22.2)	1.03	(0.53–1.80)	12/11.6	9.3	9.0	0.3	(-5.0–5.5)
2+ years	1.49	(1.06–2.05)	38/25.5	27.4	18.4	9.0	(0.3–17.7)	0.91	(0.49–1.56)	13/14.2	9.8	10.7	-0.9	(-6.2–4.4)
**Time since hip fracture for men with CCI > 0**														
0-1 months	7.91	(6.62–9.38)	132/16.7	170.0	21.5	148.5	(119.5–177.5)	15.93	(12.2–20.4)	63/4.0	158.4	9.9	148.4	(109.3–187.6)
1-3 months	3.60	(3.04–4.23)	147/40.8	76.9	21.4	55.5	(43.1–67.9)	5.22	(3.90–6.85)	52/10.0	50.6	9.7	40.9	(27.2–54.7)
3-12 months	2.09	(1.78–2.43)	162/77.7	44.2	21.2	23.0	(16.2–29.8)	2.88	(2.19–3.70)	60/20.9	28.1	9.8	18.3	(11.2–25.4)
1-2 years	1.96	(1.63–2.33)	128/65.5	40.9	20.9	20.0	(12.9–27.1)	2.43	(1.80–3.21)	49/20.2	25.4	10.4	14.9	(7.8–22.1)
2+ years	1.67	(1.33–2.07)	82/49.2	35.3	21.2	14.1	(6.5–21.8)	2.65	(1.92–3.55)	44/16.6	28.3	10.7	17.6	(9.2–26.0)

The background population for these calculations were all men with PCa in PCBaSe Sweden (Red arrows in Figure 1). The SMRs were calculated by dividing the observed number of events (obs) by the expected number of events (exp).

## Discussion

To our knowledge, this is the largest study on risk of death after hip fractures in men with PCa. A hip fracture was associated with an extra 30 deaths per 1,000 person-years among PCa men on androgen deprivation therapy (ADT) and an extra 20 deaths per 1,000 person-years among PCa men not on ADT, in comparison with the total PCa population.

We aimed to evaluate how PCa and/or ADT affect the known association between hip fracture and risk of death. Firstly, our study corroborates the previously demonstrated increase in death following a hip fracture for all men, independently of a PCa diagnosis [7]. Both the absolute risk differences and the SMRs comparing PCa men with the Swedish male population showed a similar risk of death following a hip fracture for PCa-free men and PCa men not on ADT.

The absolute risk and absolute risk differences were largest for PCa men on ADT. Thus, the largest number of extra cases of death following a hip fracture will be in PCa men on ADT. However, the current study is an observational study so we cannot disentangle whether the increased mortality among PCa men on ADT is due to ADT per se or the advanced cancer treated by ADT. It is possible that a hip fracture following ADT is just a sign of incipient end-stage disease. Moreover, fractures can also be the result of frailty not related to PCa and be a proxy for other chronic diseases [5], [7], [16]. It is also possible that rehabilitation after a hip fracture is more difficult for men with PCa on ADT than for men with PCa not on ADT or PCa-free men, so that a hip fracture is a stronger marker of overall frailty in this population.

The nationwide population-based linkable health-care registers enabled us to assess the risk of mortality following hip fracture with unprecedented precision. PCBaSe 2.0 includes almost 110,000 cases of PCa diagnosed since 1996, representing more than 96% of all new cases registered in the Swedish Cancer Register during the same period [18]. For these patients we have information on tumour characteristics at time of diagnosis, initial treatment, socio-economic status and comorbidity. The same information on hip fracture and death was available for the entire background population, which thus resulted in the ability to perform SMR calculations. The bias in the SMRs due to using general population rates, which included men with PCa, to estimate expected numbers of death was found to be of little effect [26], [28]. The influence of the treatment choice on the results should be minor since PCa risk category was adjusted for. Notwithstanding the above there may be some residual bias that cannot be accounted for. In the present study we lack information on lifestyle related factors such as smoking habits or BMI.

## Conclusions

Our findings indicate that hip fractures are associated with higher all-cause mortality in PCa men on ADT than in PCa men not on ADT or PCa-free men. The risk was especially high during the first months after the hip fracture. PCa men not on ADT and PCa-free men had similar increase in risk of dying after a hip fracture. These results are of clinical importance as they point towards the need for careful management of PCa patient on ADT when they present with a hip fracture and question whether these hip fractures can or should be prevented or treated with a rehabilitation programme that takes these increased risks into account.
